# Lack of Correlation between Aberrant p16, RAR-β2, TIMP3, ERCC1, and BRCA1 Protein Expression and Promoter Methylation in Squamous Cell Carcinoma Accompanying *Candida albicans*-Induced Inflammation

**DOI:** 10.1371/journal.pone.0159090

**Published:** 2016-07-13

**Authors:** Yui Terayama, Tetsuro Matsuura, Kiyokazu Ozaki

**Affiliations:** Laboratory of Pathology, Faculty of Pharmaceutical Sciences, Setsunan University, Osaka, Japan; University of Navarra, SPAIN

## Abstract

Hyperplastic candidiasis is characterized by thickening of the mucosal epithelia with *Candida albicans* infection with occasional progression to squamous cell carcinoma (SCC). *C*. *albicans* is a critical factor in tumor development; however, the oncogenic mechanism is unclear. We have previously produced an animal model for hyperplastic candidiasis in the rat forestomach. In the present study, we investigate whether impaired DNA methylation and associated protein expression of tumor suppressor and DNA repair genes are involved in the SCC carcinogenesis process using this hyperplastic candidiasis model. Promoter methylation and protein expression were analyzed by methylation specific PCR and immunohistochemical staining, respectively, of 5 areas in the forestomachs of alloxan-induced diabetic rats with hyperplastic candidiasis: normal squamous epithelia, squamous hyperplasia, squamous hyperplasia adjacent to SCC, squamous hyperplasia transitioning to SCC, and SCC. We observed nuclear p16 overexpression despite increases in *p16* gene promoter methylation during the carcinogenic process. *TIMP3* and *RAR-β2* promoter methylation progressed until the precancerous stage but disappeared upon malignant transformation. In comparison, TIMP3 protein expression was suppressed during carcinogenesis and RAR-β2 expression was attenuated in the cytoplasm but enhanced in nuclei. *ERCC1* and *BRCA1* promoters were not methylated at any stage; however, their protein expression disappeared beginning at hyperplasia and nuclear protein re-expression in SCC was observed only for ERCC1. These results suggest that aberrant p16, RAR-β2, TIMP3, ERCC1, and BRCA1 expression might occur that is inconsistent with the respective gene promoter methylation status, and that this overexpression might serve to promote the inflammatory carcinogenesis caused by *C*. *albicans* infection.

## Introduction

*Candida albicans* (*C*. *albicans*) is known to induce oral, esophageal, and vaginal candidiasis [1]. Severe *Candida* infections in patients are often associated with neoplastic disease [2, 3]. In addition, oral mucosa with hyperplastic candidiasis can become dysplastic from the hyperplasia and ultimately progress to carcinoma [1, 3, 4]. *C*. *albicans* has the ability to produce carcinogens such as nitrosamines; thus, the persistent infection of *C*. *albicans in vivo* acts as a promoter of oncogenesis [3, 5]. Therefore, *C*. *albicans* infection serves as a critical factor in tumor development as well as a cause of inflammation. However, little is currently known concerning the oncogenic mechanism of *C*. *albicans* infection.

Previously, we have reported that alloxan-induced diabetic rats frequently developed severe mucosal proliferative lesions with *C*. *albicans* and bacterial infection in the forestomach and that these lesions progressed to squamous cell carcinoma (SCC) [6, 7]. Additionally, we have succeeded in inducing early onset proliferative and inflammatory lesions by dosing *C*. *albicans* via ingestion in rats with hyperglycemia and have defined them as a diabetic rodent model for *C*. *albicans*-induced mucosal inflammation and proliferation [8]. These proliferative lesions are characterized by a thickening of the squamous epithelia caused by *C*. *albicans* infection-induced inflammatory changes. Notably, papillomatous proliferation or papilloma is not observed among the proliferating mucosa; instead, the hyperplastic epithelia directly progress to invasive carcinoma. The basal cells of the hyperplastic mucosa infiltrate beyond the muscularis mucosa to its submucosa in the carcinogenesis process; this invasive carcinoma represents a well-differentiated SCC with desmoplasia. As this progression is similar to that observed in oral and/or esophageal hyperplastic candidiasis in humans, we were convinced that our model might be particularly suitable to analyze the mechanism of multistep carcinogenesis in *C*. *albicans*-induced SCC.

Oncogene and tumor suppressor gene aberrations are generally associated with tumorigenesis. Epigenetic changes such as nucleotide and histone modifications play a significant role in tumorigenesis in addition to genetic changes such as mutation and deletion [9]. In oral SCC and gastric cancer, abnormal epigenetic changes are reportedly observed from the early stages of the multistep carcinogenesis process, which induce genetic abnormalities owing to the accumulation of a large number of epigenetic alterations [10–12]. The most commonly researched epigenetic mechanism in carcinogenesis studies is DNA methylation. In SCC occurring in the esophagus, oral cavity, and lung, the DNA methylation of many tumor suppressor genes (*p16*, *p15*, *CADM1*, *TIMP3*, *RAR-β2*, *RASSF1A*, *DAPK1*, and *SOCS-3*) and DNA repair genes (*ERCC1*, *BRCA1*, *XRCC1*, and *MLH1*) suppresses their protein expression and induces abnormal cell cycle and proliferative signaling leading to the accumulation of mutations [13–17]. In addition, during inflammatory carcinogenesis, *Helicobacter pylori* infection and its inflammatory response have been shown to induce aberrant DNA methylation in gastric epithelia; furthermore, the duration of the smoking period (*i*.*e*., the inflammatory trigger in smokers) is well correlated with DNA methylation alterations in the esophagas [11, 18]. Thus, it is probable that aberrant DNA methylation followed by altered protein expression might also occur in *C*. *albicans*-induced chronic inflammation-associated cancer in the forestomach in alloxan-induced diabetic rats.

In this study, we investigated the association of aberrant DNA methylation and protein expression of tumor suppressor and DNA repair genes with the process of multistep carcinogenesis using our animal model for hyperplastic candidiasis and determined the potential role such changes might play in the mechanism underlying SCC.

## Materials and Methods

### Animals and diet

WBN/Kob rats were obtained from Japan SLC, Inc. (Shizuoka, Japan). The rats were reared in a barrier-sustained animal room maintained at a temperature of 24 ± 2°C with a relative humidity of 60 ± 20%, a 12 h light/dark cycle, and ventilated at least 12 times/h. Rats were provided a standard diet and chlorinated water *ad libitum*. Rats were inspected daily by the lead investigator and personnel; the inspections included handling and body weight measurement. The specific criteria used to monitor animal health (e.g., condition of fur and skin, eyes, and excrement; absence of disordered breathing and movement, weight loss, injuries, abscess, and edema) were codified in a protocol with recommendations on possible actions. In addition, we had a protocol in place for early euthanasia endpoints for animals that became severely ill during the experiments. The study was approved by the Committee for Animal Experiments of Setsunan University.

### Experimental design

**Fig 1:**
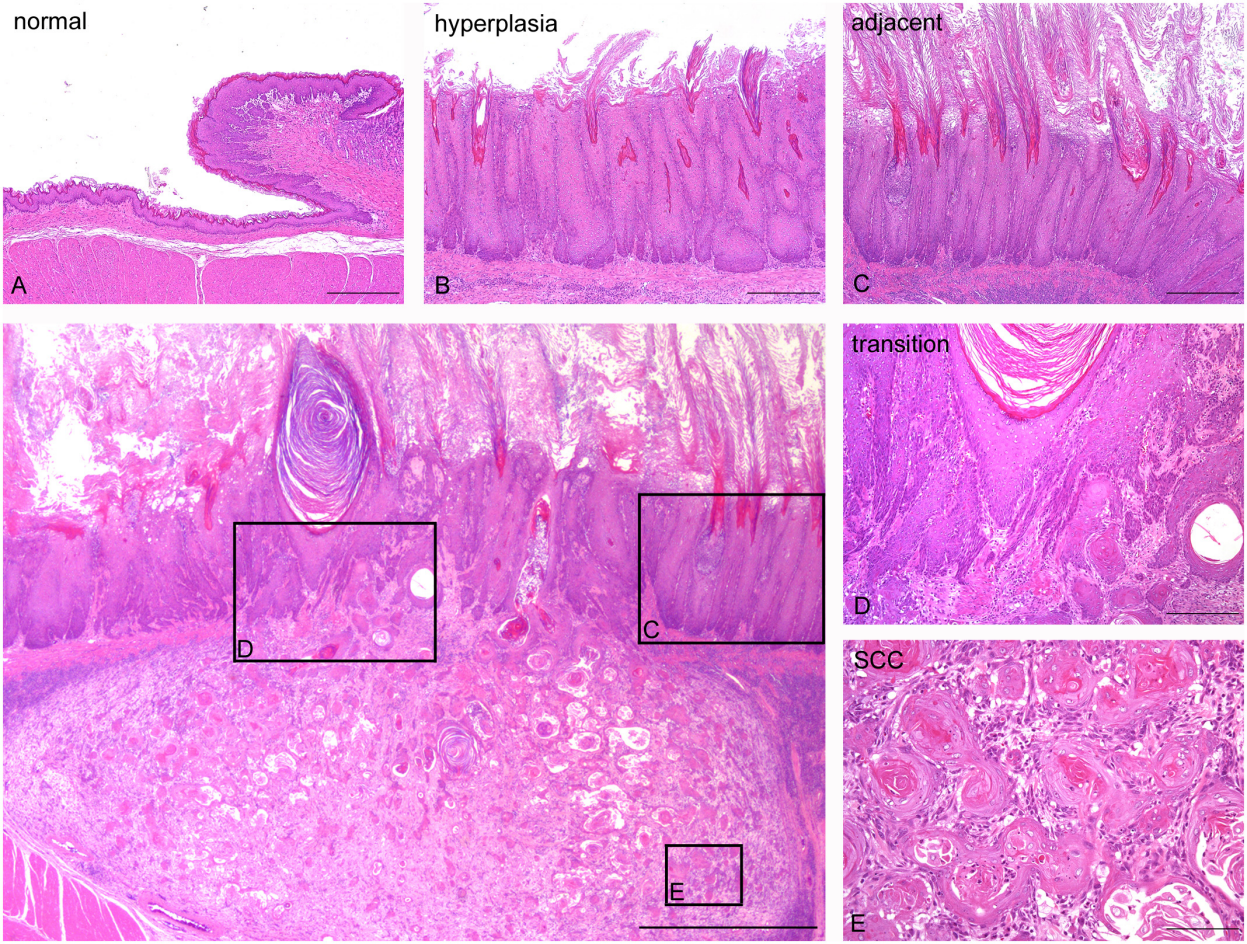
Analyzed regions of squamous epithelia in the forestomach in alloxan-induced hyperplastic candidiasis and SCC. (A) Normal epithelia (normal); (B) squamous hyperplasia (hyperplasia); (C) squamous hyperplasia adjacent to SCC (adjacent); (D) squamous hyperplasia transitioning to SCC (transition); (E) SCC; and (F) low-power magnification of (c)-(e). HE stain. Scale bar: 100 μm (A-E), 1 mm (F).

WBN/Kob rats aged 10 weeks were administered a single dose of alloxan (Sigma-Aldrich Japan, Tokyo, Japan) via the tail vein at a dosage level of 45 mg/kg (males) and 40 mg/kg (females) [8]. At 30 weeks after alloxan treatment, all rats were anesthetized via intraperitoneal injection of 40 mg/kg ketamine hydrochloride (Ketalar, Sankyo, Tokyo) and 10 mg/kg xylazine hydrochloride (Seractal, Bayer Japan, Tokyo). The organs of the 24 rats were dissected out and immersed in 10% phosphate-buffered formalin solution; the fixed organs were then embedded in paraffin. Sections (4 μm thickness) of the tissue specimens were stained with hematoxylin-eosin. We analyzed 11 cases of squamous cell hyperplasia and 6 cases of SCC (Fig 1). In addition, 6 cases of normal squamous epithelia in non-diabetic rats were used as controls. A detailed inspection of the carcinogenesis process was performed by analyzing each area exhibiting normal morphology (Fig 1A), hyperplasia (Fig 1B), hyperplasia adjacent to SCC (the area of squamous cell hyperplasia surrounding SCC) (Fig 1C and 1F), hyperplasia transitioning to SCC (the area of transition between the squamous cell hyperplasia and SCC) (Fig 1D and 1F), and SCC (Fig 1E and 1F).

### Laser capture microdissection and DNA methylation analysis

To determine the epigenetic profiles during carcinogenesis, we utilized 8 μm thick tissue sections of areas of normal, mucosal hyperplasia, mucosal hyperplasia adjacent to SCC and SCC. Approximately 4–10 regions from the each category were microdissected using a mmi CellCut (MMI Onc., Rockledge, FL, USA). DNA was extracted and bisulfite conversion was performed using an EpiTect Plus Bisulfite Kit (QIAGEN, Hilden, Germany). Methylation specific PCR (MSP) was performed using an EpiTect MSP Kit (QIAGEN). The primers used amplified the CpG islands of the tumor suppressor gene (*p16*, *p15*, *CADM1*, *TIMP3*, *RAR-β2*, *RASSF1A*, *DAPK1*, and *SOCS-3*) and DNA repair gene (*ERCC1*, *BRCA1*, *XRCC1*, and *MLH1*) promoters (S1 Table). The selected primers were used to identify a loss of protein expression resulting from methylation in a previous study [13–17]. The thermal cycling conditions were as follows: 1 cycle at 95°C for 10 min; 40 cycles of 94°C for 15 s, primer-specific annealing temperature for 30 s, and 72°C for 30 s; and 1 cycle at 72°C for 10 min (S1 Table). The PCR products were separated on 4% agarose gels and visualized under UV illumination. DNA from the normal rat forestomach was amplified by an Illustra GenomiPhi V2 DNA Amplication Kit (GE Healthcare, Little Chalfont, UK) and was used as fully unmethylated DNA. A portion of the unmethylated DNA was then methylated by *Sss*I methylase (New England Biolabs, Ipswich, MA, USA) and was used as fully methylated DNA.

### Immunohistochemical Analysis

Immunohistochemical analysis was performed on those proteins (p16, RAR-β2, TIMP3, ERCC1, and BRCA1) whose promoter methylation status was judged as being abnormal by MSP. Immunohistochemical confirmation of protein expression level was conducted on representative sections. The sections were treated following the protocol shown in S2 Table to retrieve the antigens. Incubation was carried out overnight at 4°C using the respective primary antibody (S2 Table). The slides were subsequently treated for 30 min at room temperature with the secondary antibody (S2 Table) and incubated in a diaminobenzidine solution. As a negative control, mouse or rabbit isotype immunoglobulin, diluted to the same concentration, was substituted for the primary antibody. The evaluation methods in squamous epithelia were as follows: p16 nuclear protein expression was classified into four grades as follows: −, negative; +, weak expression; ++, moderate expression; +++, strong expression. RAR-β2 nuclear protein expression was classified into four grades as follows: −, negative; +, weak expression; ++, moderate expression; +++, strong expression, and its cytoplasmic expression into three grades as follows: −, negative; +, weak expression; ++, moderate expression. TIMP3 nuclear protein expression was classified into two grades as follows: −, negative; +, positive and its cytoplasmic protein expression within and external to the perinuclear cytoplasm was classified into three grades as follows: −, negative; +, weak expression; ++, moderate expression. The expression of the ERCC1 and BRCA1 nuclear proteins were classified into two grades as follows: −, negative; +, positive.

### Statistical analysis

The Mann-Whitney *U* test was used for statistical analysis of the immunohistochemical findings. When the calculated *p* value was less than 0.05, the difference was considered statistically significant. Statistical analysis was performed using the StatMate III program (ATMS, Tokyo, Japan).

## Results

### *p16* promoter methylation increased yet p16 nuclear protein was progressively overexpressed during the carcinogenic process

**Fig 2:**
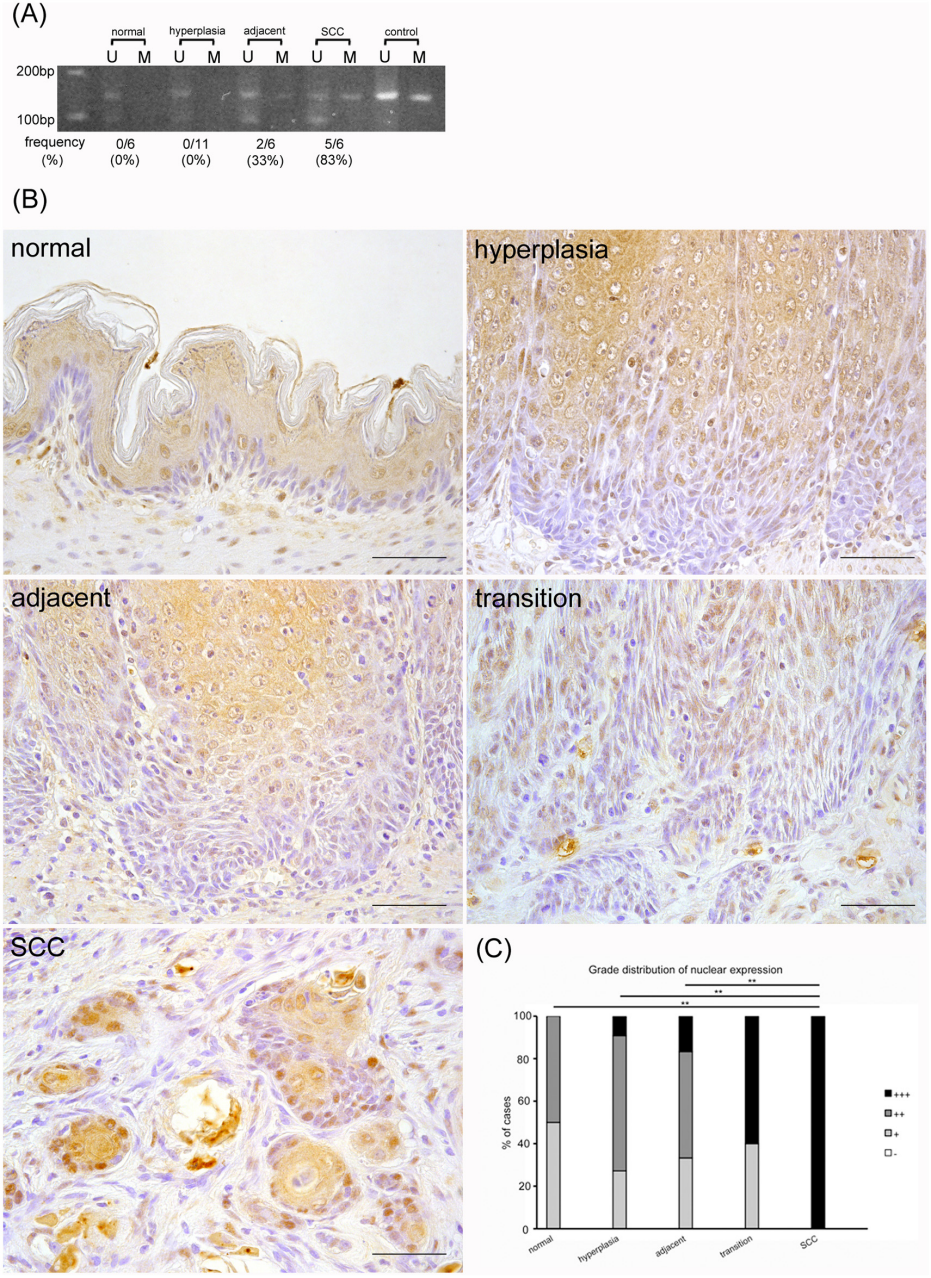
Nuclear p16 protein is progressively overexpressed in the carcinogenic process despite increased *p16* promoter methylation. (A) MSP analysis of *p16*. U: PCR product amplified by unmethylated-specific primers, M: PCR product amplified by methylated-specific primers. (B) In immunohistochemical analysis, the positivity of the nuclei of basal cells gradually increases in accordance with the carcinogenic process. (C) Grade distribution of p16 nuclear expression. **p<0.01. Scale bar: 50 μm.

The frequency of rats showing *p16* promoter methylation progressively increased as follows: 0% in normal epithelia and hyperplasia, 33% in hyperplasia adjacent to SCC, and 83% in SCC (Fig 2A). The expression of the p16 protein differed depending on cellular differentiation status, with the upper layer of the squamous epithelia showing high levels of staining compared to the lower layer of normal epithelia (Fig 2B and 2C). Almost all of the nuclei and cytoplasm of the differentiated cells showed a positive reaction for p16; however, in the basal cells, only a few nuclei were positive for p16. In comparison, the positivity of the basal cell nuclei gradually advanced throughout the carcinogenesis process. In SCC, all of the nuclei of the basal cell layer were strongly stained. However, the nuclear and cytoplasmic expression in the differentiated cells of the upper layer was not changed from hyperplasia to SCC. These results suggest that although the promoter methylation of *p16* was increased, the nuclear protein in the basal cells was nevertheless progressively overexpressed during the carcinogenic process.

### *TIMP3* promoter methylation increased until the precancerous stage but disappeared upon malignant transformation; nevertheless, TIMP3 protein expression was similarly suppressed during the carcinogenic process

**Fig 3:**
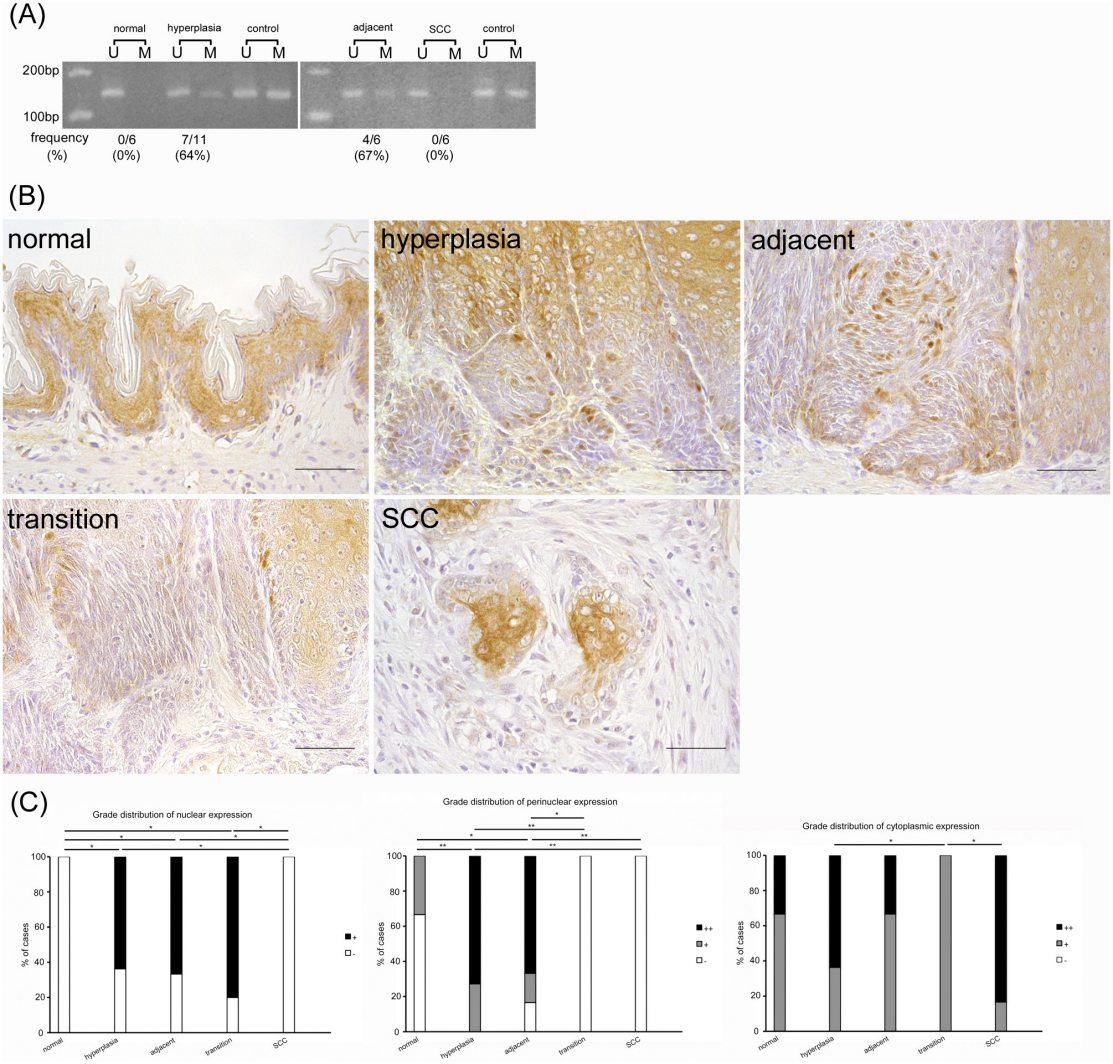
*TIMP3* promoter methylation increases until the precancerous stage but clearly disappears along with malignant transformation whereas protein expression is suppressed in the carcinogenic process. (A) MSP analysis of *TIMP3*. U: PCR product amplified by unmethylated-specific primers, M: PCR product amplified by methylated-specific primers. (B) In immunohistochemical analysis, nuclear and perinuclear cytoplasmic TIMP3 protein expression is suppressed in the carcinogenic process whereas cytoplasmic protein expression is observed throughout. (C) Grade distribution of TIMP3 nuclear, perinuclear, and cytoplasmic expression. *p<0.05, **p<0.01. Scale bar: 50 μm.

The frequency of rats showing *TIMP3* promoter methylation was 0% in normal squamous epithelia and rapidly increased in hyperplasia (64%) and hyperplasia adjacent to SCC (67%); however the methylation was clearly lost in SCC (0%) (Fig 3A). Nuclear TIMP3 protein expression was also negative in normal epithelia and rapidly advanced in the basal cells in hyperplasia and hyperplasia adjacent to SCC, but was completely lost in SCC (Fig 3B and 3C). In comparison, TIMP3 in the perinuclear cytoplasm was sporadically observed in normal epithelia and was increased in hyperplasia and hyperplasia adjacent to SCC but was completely lost in hyperplasia transition to SCC and SCC. Cytoplasmic protein expression except within the perinuclear cytoplasm was observed throughout the carcinogenic process including within normal epithelia and was most prominent in differentiated cells. However, there was no apparent tendency in cytoplasmic staining that occurred during the carcinogenic process. These results indicate that *TIMP3* promoter methylation increased until the precancerous stage yet clearly disappeared along with malignant transformation and that the associated protein expression was similarly suppressed during the carcinogenic process.

### *RAR-β2* promoter methylation progressed until the precancerous stage but disappeared upon malignant transformation whereas RAR-β2 protein expression declined in the cytoplasm but increased in the nucleus during the carcinogenic process

**Fig 4:**
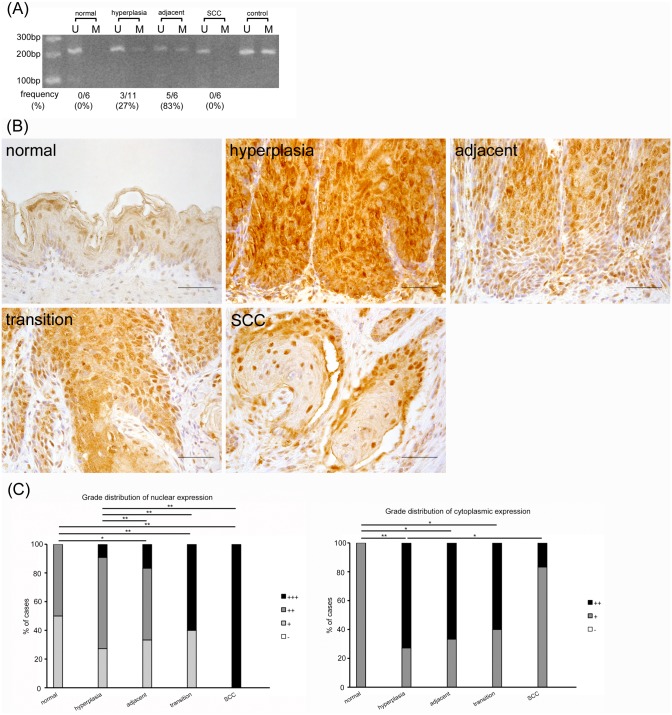
*RAR-β2* promoter methylation progresses until the precancerous stage but disappears upon malignant transformation. In contrast, its protein expression declines in the cytoplasm but increases in the nucleus during the carcinogenic process. (A) MSP analysis of *RAR-β2*. U: PCR product amplified by unmethylated-specific primers, M: PCR product amplified by methylated-specific primers. (B) In immunohistochemical analysis, RAR-β2 nuclear protein expression is gradually enhanced and cytoplasmic protein expression is also advanced in hyperplasia, but is gradually reduced during the carcinogenic process. (C) Grade distribution of nuclear and cytoplasmic expression. *p<0.05, **p<0.01. Scale bar: 50 μm.

The frequency of rats showing *RAR-β2* promoter methylation was 0% in normal epithelia and gradually increased in hyperplasia (27%) and hyperplasia adjacent to SCC (83%) but was rapidly lost in SCC (0%) (Fig 4A). In comparison, RAR-β2 protein expression in nuclei was observed only slightly in normal epithelium and was gradually enhanced during the carcinogenic process. All SCC samples exhibited strongly stained nuclei in all layers (Fig 4B and 4C). Cytoplasmic protein expression also progressed in hyperplasia but was gradually reduced during the carcinogenic process. These results suggest that *RAR-β2* promoter methylation increased until the precancerous stage but disappeared upon malignant transformation. On the other hand, RAR-β2 protein expression declined in the cytoplasm but increased in the nucleus during carcinogenesis.

### *ERCC1* and *BRCA1* promoters were not methylated during the carcinogenesis process and their proteins disappeared in the early stage; however, ERCC1 protein reappeared in the nucleus concomitant with progression of the carcinogenic process

**Fig 5:**
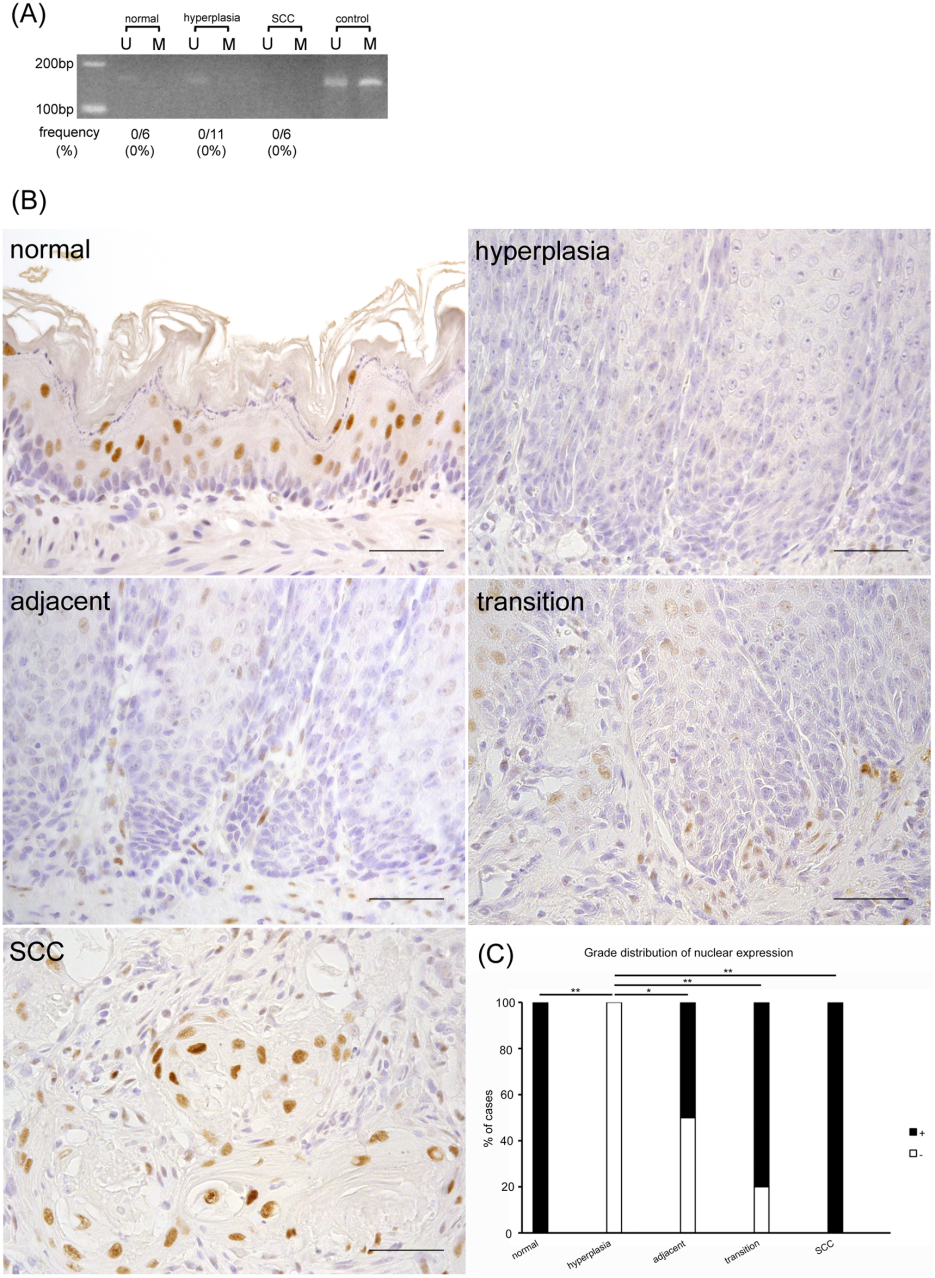
*ERCC1* promoter is not methylated in carcinogenesis process, and its protein disappears in early stage, but its protein reappears in the nucleus with progression of carcinogenic process. (A) MSP analysis of *ERCC1*. U: PCR product amplified by unmethylated-specific primers, M: PCR product amplified by methylated-specific primers. (B) In immunohistochemical analysis, ERCC1 protein expression is detected in the nuclei of normal, and once disappears in squamous hyperplasia, but gradually reappears with progression of carcinogenesis process. (C) Grade distribution of nuclear expression. **p<0.01. Scale bar: 50 μm.

**Fig 6:**
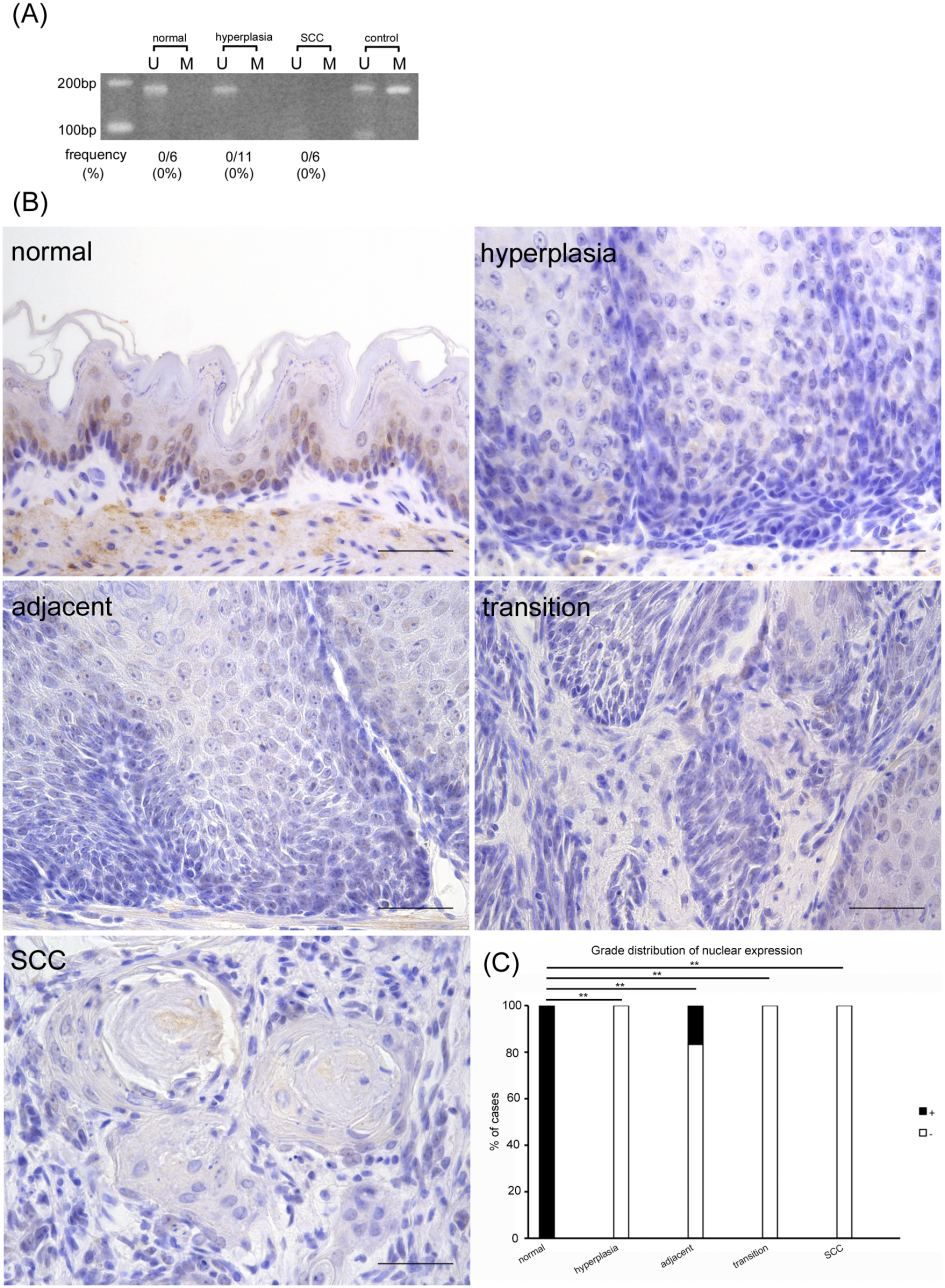
The *BRCA1* promoter is not methylated during the carcinogenic process and its protein disappears at an early stage. (A) MSP analysis of *BRCA1*. U: PCR product amplified by unmethylated-specific primers, M: PCR product amplified by methylated-specific primers. (B) BRCA1 protein is expressed in nuclei of normal tissue but drastically diminishes from hyperplasia to SCC. (C) Grade distribution of nuclear expression. **p<0.01. Scale bar: 50 μm.

*ERCC1* and *BRCA1* promoters were not methylated at any stage; nevertheless their unmethylated bands were detected in normal epithelia but disappeared during the carcinogenesis process (Figs 5A and 6A). In comparison, the ERCC1 protein was expressed in the nuclei of normal epithelia and subsequently disappeared in squamous hyperplasia but gradually reappeared in the nuclei on the differentiated cells of the suprabasal layer concomitant with the progression of the carcinogenesis process (Fig 5B and 5C). Furthermore, the BRCA1 protein was expressed in the nuclei of normal epithelia but was drastically diminished from the stages of hyperplasia to SCC (Fig 6B and 6C). In contrast, the cytoplasmic expression of ERCC1 and BRCA1 proteins was negative in all stages. These findings suggest that the *ERCC1* and *BRCA1* promoters were not methylated during the carcinogenesis process and that their proteins disappeared at an early stage, but that the ERCC1 protein reappeared in the nucleus with the progression of carcinogenesis.

## Discussion

*p16* is a tumor suppressor gene whose product is involved in regulation of the cell cycle and plays an important role in cellular senescence, the induction of apoptosis, and cell infiltration via mechanisms other than regulation of the cell cycle [19]. Hypermethylation of the *p16* gene promoter is reported in many tumors such as oral, esophageal, and lung squamous cell carcinoma [9, 13, 17, 20]. In almost all cases, *p16* promoter hypermethylation suppresses its protein expression. In the present study, *p16* promoter methylation proceeded in accordance with the carcinogenesis process as has been shown in previous reports in humans [9, 13, 17, 20]. However, its protein expression was not suppressed in the nuclei of SCC. p16 protein overexpression reportedly occurs in cancer owing to mutation of the *CDKN2A* gene or feedback control caused by loss of the *Rb* gene [19, 21]. The cytoplasmic overexpression of the p16 protein is also reported to be associated with formation of the p16/CDK4 complex and p16 binding with transmembrane proteins [22, 23]. In *C*. *albicans*-induced chronic inflammation-associated cancer, it is probable that p16 protein overexpression might be caused by alternative pathways such as *CDKN2A* gene mutation or loss of the *Rb* gene irrespective of *p16* promoter hypermethylation.

TIMP3 is related to tumor development and in particular has been shown to antagonize the activity of matrix metalloproteinases as well as to inhibit tumor growth, angiogenesis, invasion, and metastasis [24, 25]. *TIMP3* promoter methylation is observed in many tumors such as esophageal, gastric, and thyroid cancer, and causes loss of TIMP3 protein expression [24, 26, 27]. However, the down-regulation of TIMP3 protein not resulting from *TIMP3* promoter hypermethylation has also been reported in pancreatic adenocarcinoma; this phenomenon is also known to be associated with microRNA and the loss of heterozygosity [28–31]. In this study, we identified that the *TIMP3* promoter was methylated until the precancerous stage but was then demethylated with associated diminished TIMP3 protein expression. In addition, gradual reduction of TIMP3 protein expression was present concomitant with progression of the carcinogenesis process. These data suggest that loss of TIMP3 protein expression might not be directly associated with promoter methylation status as has been found in previous reports, but might instead be related to neoplastic transformation consequential to *C*. *albicans*-induced chronic inflammation-associated cancer.

RAR-β2 reportedly leads to the inhibition of cell growth and induction of apoptosis and its gene and mRNA expression disappears because of methylation of the DNA promoter region in SCC and dysplasia of the esophagus and oral mucosa [32–34]. By immunohistochemical analysis, significant reduction of nuclear and cytoplasmic RAR-β2 protein expression has been reported in malignant tumors such as oral squamous cell carcinoma and thyroid carcinoma; however, up-regulation such as observed in our study has rarely been reported in malignant tumors [33, 35–37]. On the other hand, RAR-β2 protein expression in the nuclei and cytoplasm is found in benign lesions and low-grade cancers such as benign thyroid tumor, oral dysplasia, prostate hyperplasia, and prostatic intraepithelial neoplasm [33, 36, 38]. The SCC examined in our study is a well-differentiated low-grade cancer with a low metastatic rate and good prognosis [7]. These findings suggest that the RAR-β2 protein expression observed in nuclei and the cytoplasm might be related to the low-grade malignancy of the tumor. Furthermore, we observed that the cytoplasmic protein expression suddenly elevated in hyperplasia and then declined to the level comparable to that of normal epithelia in SCC. Similar changes have been reported in prostate hyperplasia and cancer, wherein it was suggested that a distinct relationship existed between RAR-β signaling and prostate hyperplasia [38]. Thus, it is probable that up-regulation of RAR-β signaling is associated with squamous hyperplasia.

*ERCC1* is a DNA repair gene whose protein product plays an important role in nucleotide excision repair; both its protein and mRNA expression is reduced in colon cancer and glioma [39–41]. On the other hand, the ERCC1 protein is known to be overexpressed in human head and neck squamous cell carcinoma and esophageal carcinoma [42, 43]. In this study, since its protein expression disappeared upon hyperplasia irrespective of promoter methylation, the underlying causative factor might involve gene mutation, degradation of the proteins by ROS, or epigenetic change other than promoter methylation [40]. Notably, with the progression of the carcinogenic process, ERCC1 protein expression gradually re-emerged in suprabasal nuclei with concomitant loss of both the unmethylated and methylated bands from the promoter regions. The cause of the loss of the unmethylated bands in our results is unclear. Our findings suggest that an abnormality in the promoter region might enhance ERCC1 protein expression in *C*. *albicans*-induced cancer.

*BRCA1* is a tumor suppression gene whose protein product is involved in the maintenance of DNA homeostasis via multiple mechanisms such as DNA repair, transcription, cell cycle, and apoptosis [44]. Gene mutation and loss of protein expression reportedly exist in breast and ovarian cancer and the loss and dysfunction of the BRCA1 protein induced by *BRCA1* promoter hypermethylation have been identified in breast and ovarian cancer [44–46]. In addition, BRCA1 protein and mRNA expression varies from high to low levels in human esophagus squamous cell carcinoma [47]. In this study, BRCA1 protein expression was lost in hyperplasia, which is an early stage of the carcinogenic process, despite the lack of promoter methylation. It is likely that BRCA1 inactivation might be related to the early stage of neoplastic transformation irrespective of promoter methylation status.

In human esophageal SCC, the protein expression of p16, RAR-β2, and TIMP3 is decreased, and ERCC1 and BRCA1 protein expression varies [20, 26, 32, 43, 47]. In our study, the change in TIMP3, ERCC1, and BRCA1 levels in SCC induced by *C*. *albicans* was mostly consistent with that in human SCC, whereas the high expression of p16 and RAR-β2 did not correspond to the low expression observed in human SCC. However, p16 protein overexpression has been observed in human esophageal SCC associated with infection by papilloma virus [48]. Because chronic inflammation caused by *C*. *albicans* infection is involved in p16 protein overexpression, it is probable that similar to human papillomavirus, *C*. *albicans* directly influences p16 expression. RAR-β2 protein expression is reduced in human prostate cancer with high malignancy [38]. Because the SCC in our study is a well-differentiated and low-grade cancer with a low metastasis rate [7], the relative lack of malignancy of the tumor may affect the RAR-β2 protein expression.

In SCC induced by *C*. *albicans* infection, DNA methylation of particular CpG islands in the promoter regions of p16, TIMP3, ERCC1, BRCA1, and RAR-β2 may not affect protein expression. To our knowledge, only a few reports have demonstrated a relative lack of correlation of methylation patterns with protein levels [30]. In addition to DNA methylation, RNA levels are regulated by microRNA, and protein levels are regulated at the level of protein stability [49, 50]. RNA and protein expression of p16, TIMP3, ERCC1, and BRCA1 is decreased by a reduction of microRNA levels [31, 40, 51, 52]. In addition, p16 protein stability is regulated by ARF [53], and RAR-β2 protein expression is involved in up-regulation of retinoic acid [54]. That is, it is probable that factors other than DNA methylation also affected the protein expression and were involved in tumorigenesis in our study. Further studies are needed to determine the factors involved in this lack of correlation to analyze the oncogenic mechanism of *C*. *albicans*-induced chronic inflammation-associated cancer.

## Conclusion

**Fig 7:**
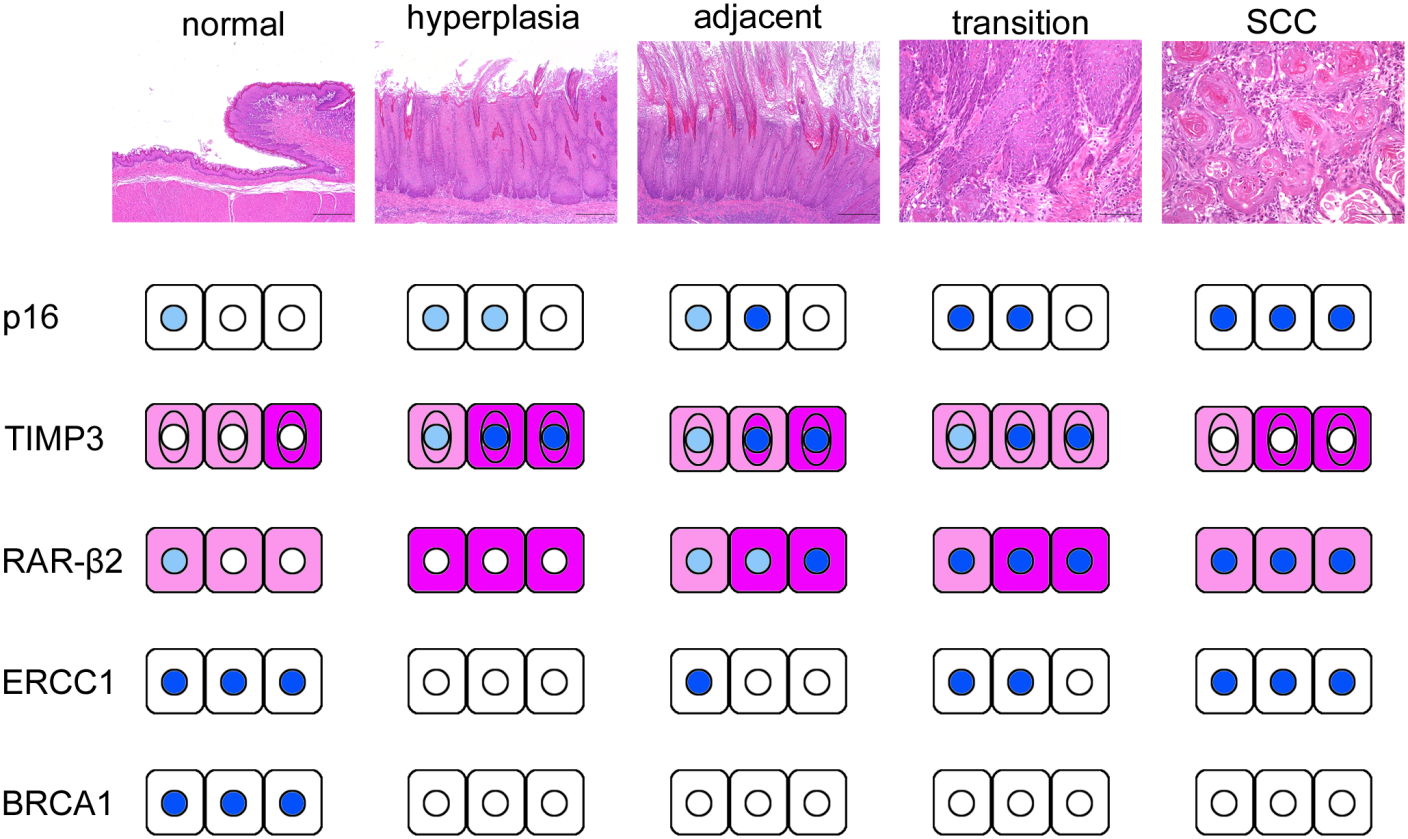
Schematic representation of alterations of protein expression during SCC carcinogenesis associated with chronic inflammation and *C*. *albicans* infection.

We demonstrated that altered expression of p16, RAR-β2, TIMP3, BRCA1 and ERCC1 was associated with the carcinogenic process of SCC subsequent to chronic inflammation and *C*. *albicans* infection. The protein expression of BRCA1 and ERCC1 was lost in hyperplasia; subsequently, aberrant accumulation of p16 and RAR-β2 proteins occurred along with progression of the carcinogenesis process. Finally, TIMP3 protein expression was reduced in SCC (Fig 7). However, the promoter hypermethylation status of *p16*, *RAR-β2*, *TIMP3*, *BRCA1*, and *ERCC1* did not correlate with the loss of protein expression and thus is not likely to be the cause thereof. The present results suggest that aberrant protein expression of p16, RAR-β2, TIMP3, BRCA1, and ERCC1 might occur regardless of promoter methylation and might promote the process of inflammatory carcinogenesis caused by *C*. *albicans* infection.

## Supporting Information

S1 Table Primer sequences for methylation-specific PCR. (PDF)

S2 Table Antibodies used in this study. (PDF)
