# Supplementary material for: Lack of Correlation between Aberrant p16, RAR-β2, TIMP3, ERCC1, and BRCA1 Protein Expression and Promoter Methylation in Squamous Cell Carcinoma Accompanying Candida albicans-Induced Inflammation
Source: PLoS One. 2016 Jul 13;11(7):e0159090. doi: 10.1371/journal.pone.0159090 (PMC4943641; doi:10.1371/journal.pone.0159090)
Supplement: S1 Table — (PDF) [file pone.0159090.s001.pdf]

S1 Table 1. Primer sequences for methylation specific PCR

Gene	M/U	Primer sequence (5' → 3')	Length (bp) position	Annealing temperature (℃)
p16	M	F: aattcgaggagagcgattcg	133	52
		R: gaccgaaaatattcgaaacg		
	U	F: gtgaatttgaggagagtgatttg	122	52
		R: caaccaaaaatattcaaaacatt		
p15	M	F: ggaggattattttttgttacggatc	138	52
		R: gacgttaaaatctacgccgac		
	U	F: gaggattatttttgttatggattgg	138	52
		R: taacaacattaaaatctacaccaac		
CADM1	M	F: gaggtaggtgttcgatatggc	150	52
		R: cctataaaaatcaataccgcgac		
	U	F: ggtaggtgtttgatatggtga	150	52
		R: cacctataaaaatcaataccacaac		
TIMP3	M	F: tttcggaggttatttattggtttc	139	52
		R: cgaatttaaccgtacactatacacg		
	U	F: tttttggaggttatttattggtttt	140	52
		R: caaatttaaccatacactatacacaat		
RAR-β2	M	F: ggttgggaaaaagattaatagtttac	222	55
		R: ctcctacaacatacaaaaaaacgaa		
	U	F: tgggaaaaagattaatagtttatgt	219	55
		R: ctcctacaacatacaaaaaaacaaa		
RASSF1A	M	F: tttgatcgggttatgtcggc	169	55
		R: gataaccacgacccgaaacg		
	U	F: tttgattgggttatgttggt	169	55
		R: aataaccacaacccaaaaca		
DAPK1	M	F: ttcgagattaggatcgagttc	194	55
		R: caatcataataaactatcacgcc		
	U	F: aggttttgagattaggattgagttt	200	55
		R: cacaatcataataaactatcacacc		
SOCS3	M	F: ggattttattggagtgtcgtaatc	153	55
		R: aatacgtaaattcttaatccccgac		
	U	F: ggattttattggagtgttgtaattg	153	55
		R: aatacataaattcttaatccccaac		
ERCC1	M	F: tttaggattatagagagtagcgcga	166	55
		R: caaaaaaaataaaaacgatacaacg		
	U	F: tttaggattatagagagtagtgtga	164	55
		R: aaaaaaataaaaacaatacaacacc		
BRCA1	M	F: ggtttttttcggtatttaggtttc	174	55
		R: ttaattccgctccaaacgtt		
	U	F: ggttttttttggtatttaggttttg	179	55
		R: aaactttaattccactccaaacatt		
XRCC1	M	F: cgtagaggtttaatcgagtatgc	128	55
		R: cccgaaaaaaataaaaaaaatacg		
	U	F: tgtagaggtttaattgagtatgtgt	129	55
		R: ccccaaaaaaaaataaaaaaaataca		
MLH1	M	F: cgtgaattttgacgcgtaagcgc	175	55
		R: ccttcaccttaaaccgaccgccg		
	U	F: ggattgtgaattttgatgtgtaagtgt	184	55
		R: ttcttccttcaccttaaaccaacca		
