# Supplementary material for: Lack of Correlation between Aberrant p16, RAR-β2, TIMP3, ERCC1, and BRCA1 Protein Expression and Promoter Methylation in Squamous Cell Carcinoma Accompanying Candida albicans-Induced Inflammation
Source: PLoS One. 2016 Jul 13;11(7):e0159090. doi: 10.1371/journal.pone.0159090 (PMC4943641; doi:10.1371/journal.pone.0159090)
Supplement: S2 Table — (PDF) [file pone.0159090.s002.pdf]

S2 Table 2. Antibodies used in this study

Primary antibody	Source	Antigen retrieval	Time	Dilution	Secondary antibody
p16	Santa Cruz	pepsin	20 min	1:50	Histofine simple stain rat MAX-PO (M)
TIMP3	Proteintech	–		1:200	Histofine simple stain rat MAX-PO (Rb)
RAR-β2	abcam	10 mM citrate buffer (pH 6.0)	10 min	1:50	Histofine simple stain rat MAX-PO (Rb)
ERCC1	Santa Cruz	10 mM citrate buffer (pH 6.0)	10 min	1:800	Histofine simple stain rat MAX-PO (M)
BRCA1	Santa Cruz	1 mM EDTA buffer (pH 8.0)	10 min	1:200	Histofine simple stain rat MAX-PO (Rb)
